# Designing technology tools to support engagement in mindfulness-based interventions: an analysis of teacher and student experiences

**DOI:** 10.1177/2055207619868550

**Published:** 2019-08-25

**Authors:** Christine E. Parsons, Kasper L. Jensen, Andreas Roepstorff, Lone O. Fjorback, Conor Linehan

**Affiliations:** 1 Interacting Minds Center, Department of Clinical Medicine, Aarhus University, Denmark; 2 School of Engineering, Aarhus University, Denmark; 3 School of Communication and Culture, Aarhus University, Denmark; 4 Danish Center for Mindfulness, Aarhus University, Denmark; 5 School of Applied Psychology, University College Cork, Republic of Ireland

**Keywords:** Mindfulness, smartphone monitoring, meditation practice, treatment engagement

## Abstract

Standard mindfulness-based interventions have significant at-home assignments of formal mindfulness practice as a key component. Engagement with formal home practice has been correlated with treatment outcomes, but participants often complete less than the assigned amounts. Here, we explore the requirements for technology tools that can support and encourage home practice, in a way that is appropriate and consistent with the core principles of mindfulness-based interventions. Interviews were held with a group of five highly experienced mindfulness teachers and a group of five participants who had previously completed an eight-week course. Data was subjected to thematic analysis. A key finding was that providing teachers with information on how students practice could support communication around difficulties with home practice. We also identified questions around the appropriateness of adapting the course in response to participant difficulties and participant preferences. Both teachers and students made numerous suggestions for ways to augment their training using technology, such as via practice reminders and provision of teacher-specific content. Finally, a major design issue for technology developers is how to support participants in reflecting on their experiences of mindfulness practice, and subsequent learning, but not to critically evaluate their practice.

## Introduction

Standard eight-week mindfulness courses, as for other psychological therapies, require participants to complete at-home assignments, in addition to group sessions. At-home assignments provide opportunities to practice new skills, test new ideas, and generalise learning outside of sessions with the therapist.^1^ For mindfulness-based stress reduction (MBSR) and mindfulness-based cognitive therapy (MBCT), at-home assignments consist of both formal and informal practices. Formal practice includes the Body Scan (typically a lying down meditation, focused on sensations in the body), yoga, and sitting meditations, generally supported by audio guides, and consists of around 45 minutes per day over the eight-week course. Along with shorter ‘informal’ practice (e.g. mindfulness in everyday routines, such as chopping vegetables), the longer formal practice is presented as a core part of the course. Practice is emphasised as one of the key means for people to become aware of, and relate differently, to mental habits.^2^

Completion of home practice is often a challenge for participants, similar to home assignments in other psychological therapies.^3^ Home practice requires participants to expose themselves to difficult subjective experiences, which they may have previously avoided.^2^ Practice has been described as ‘the slow, disciplined work of digging trenches, of working in the vineyards, of bucketing out a pond. [As] the work of moments and the work of a lifetime, all wrapped into one’^4^ (p. 111). Mindfulness-based interventions strongly emphasise experiential learning: participants are encouraged to ‘try it out’.

Cognitive behaviour therapy (CBT) is the most studied psychological treatment,^5^ and like MBSR and MBCT, it has home assignments as a central component. Home assignments can take many formats in CBT, including symptom logs, self-reflective journals, and specific, structured activities like exposure and other behavioural experiments. For example, a patient might be asked to schedule an activity associated with enjoyment or accomplishment (for a review, see Kazantzis et al.^6^). The extent to which patients complete their CBT home assignments is related to the benefit they receive from treatment (for meta-analyses see Kazantzis et al.^6,7^). These meta-analyses indicate that there is a small to moderate association between home assignment completion and treatment outcomes. That is, completing more home assignments is associated with better outcomes. The positive associations between home assignment completion and outcome hold, even when attendance at CBT treatment is taken into account, along with initial symptom severity.^8^

For MBSR and MBCT, a recent meta-analysis of 48 studies found that participants reported completing a significant amount of home practice (around 30 min a day, 6 days per week on average), but less than the recommended 45 min.^9^ There was a small to moderate association between home assignment completion and treatment benefit. Furthermore, the amount of practices that participants reported doing was highly variable: some reported practising even more than the assigned amounts and some did far less.^10^

### What are the difficulties faced by participants?

Given the importance of practice, it is crucial to understand the obstacles that participants face in its completion. A number of qualitative studies have emerged that describe some of the obstacles. Lack of time is perhaps the most frequently discussed issue.^11^ For instance, in one feasibility study of MBCT, finding a time during the day, particularly among those with external employment, was the main challenge described.^12^ A second study reported that being unemployed predicted greater engagement with home practice.^11^ The authors argued that participants with unrestricted time during the day were better able to complete their practices. Other work has described additional barriers to practice including impatience, boredom and fatigue; scepticism regarding the value of mindfulness training; the notion that mindfulness is ‘self-indulgent’; negative affective states (e.g. stress, anxiety, depression, self-criticism) and the requirement for forced breaks from routine.^13^ Finally, a study of participants 12–18 months after completing an MBCT course found that participants required reminders of the practices they had completed, and indeed reminders of the benefits of practice.^14^

One major issue in our understanding of how participants practice at home, and how this impacts on outcomes, relates to the way in which practice is typically recorded.^9^ Studies to date have relied almost entirely on participants’ retrospective, subjective, one-time self-report ratings of home practice completion. The problems with retrospective self-report are numerous, and include memory lapses, socially desirable responding, and inaccurate recall, as well as loss of paper diaries.^15^ These problems may be compounded where participants face mental health difficulties (e.g. conditions such as depression can negatively impact memory^16^), which is often the case in mindfulness-based interventions.

### Technology in mindfulness-based interventions

Technology provides a means to address this practice measurement limitation. For example, participants’ home practice guides can be played via a website or app on a mobile device, which can then log listening time. This could enable monitoring of formal home practice completion by both the participant and teacher in real time over the course of a mindfulness-based intervention. Several recent studies have used technology-based methods, such as app usage data, or web portals, to record participants’ practice time (a measure of the use of audio guides for practice) during mindfulness training. However, these studies have delivered their own mindfulness training courses, rather than using the two standardised formats, MBSR or MBCT. Often these training courses do not include a face-to-face group and have no human teacher, relying instead on automated and self-guided standalone programmes, which differ substantially from standard formats. For instance, one study using a mindfulness app with cancer patients, assigned 15 min of home practice to participants, substantially less than the 45 min assigned in MBCT or MBSR. Just over half of the patients continued to use the mindfulness app consistently until week 10, completing a median number of exercises of four at week 1, dropping to a median of two at week 10.^17^ Another study used an iPad app to record listening time during a six-week mindfulness course, again with reduced home practice requirements compared to MBSR or MBCT.^18^ Participants listened to around 23 min per day according to the app, with a drop to 16 min per day after the end of the six-week course.

Mindfulness apps are now widely available, and are popular among the general population. However, there has been substantially less empirical testing of mindfulness apps compared to CBT-based apps.^19^ Commercial mindfulness apps typically provide meditation guides, rather than full, evidence-based standardised training programmes. For instance, one review of the available ‘mindfulness’ apps identified more than 700 apps on the iTunes store. However, the majority offered standalone guided meditations, timers, or reminders only.^20^ A small minority provided actual mindfulness training content or education, and only one was supported by empirical evidence. Consistent with this, a recent review of the 16 most popular iPhone meditation apps reported that the primary function of most was to provide guided meditations. None of these were based explicitly on MBSR or MBCT.^21^

### Current study

In this study, we hope to clarify the necessary features for mobile technology tools designed to support formal home practice for participants in MBSR and MBCT courses. Specifically, we aim to understand the requirements of participants and teachers for mobile technology tools that can support participants in face-to-face MBSR and MBCT courses. While studies to date have focused on the experiences of course participants, we also sought the perspectives of experienced mindfulness teachers, who bring a wealth of understanding about the course principles and participant challenges. We therefore interviewed mindfulness teachers and past course participants about i) their experiences with home practice and ii) how technology should be designed to support engagement with the course.

## Method

### Participants

Two semi-structured group interviews were conducted with individuals who had completed MBSR courses previously, and with experienced MBSR teachers. While we focused on MBSR course teachers and participants, we note that MBCT has the same schedule of home practice,^22^ and the two courses are closely related. The interview with course participants (students) took place in March, and the teacher interview in June 2017.

Five participants (1 male, 4 female, median age = 42 years) who had previously taken part in an eight-week standard format MBSR courses at the Danish Center for Mindfulness, Aarhus University took part in the interviews. Participants attended these MBSR courses for a range of physical and psychological health issues (three for general stress, one for chronic back pain, one for head injury). Participants were selected from those attending ‘booster’ class sessions at the centre and who were available to attend on the scheduled day. Booster sessions are offered as a means to support ongoing mindfulness practice, and people attend for a variety of reasons. For example, some students want to ‘deepen’ their practice further. Other students notice a drop-off in their practice and want to re-start.^14^ The sample selected is typical of the age range and gender breakdown of those who attend courses at this centre.^23^

Five teachers participated (1 male, 4 female, median age = 50), all affiliated with the Danish Center for Mindfulness. All were trained to international standards in delivering MBSR. Each teacher had extensive experience delivering MBSR courses (median number of years teaching MBSR: 11 years, range 3–12). The teachers also reported extensive experience practising mindfulness meditation themselves (median years = 17, range 11–25).

### Materials

Semi-structured interview schedules were used to guide discussion in both participant groups. The composition of these sessions was influenced by the Experience-Centered Design framework.^24^ Within this framework, finding useful technological solutions requires us to better understand the participants’ values and experiences in engaging in the target activity more generally. Interviews are not only about technology and attitudes to technology, but more broadly consider participant experiences. Therefore, the interview schedules asked different questions of the two groups, but both were designed to encourage participants to reflect upon and discuss their experiences either in completing or teaching MBSR courses. Both groups were told that we were interested in designing technology tools to support participants in engaging with the MBSR course. Teachers were asked what they currently do to encourage practices, whether there is information that would be helpful in their teaching but which is currently inaccessible, and whether they see any conflicts between the goals and processes of mindfulness training and the idea of technological monitoring. Students were asked about how they approached their mindfulness practices, any issues they encountered, whether they used any technological tools or other support for their practice, and how they might feel about monitoring approaches.

### Procedure

The same moderator ran both interview groups. The interview time was approximately 2.5 h for each group. Interviews were conducted in a room at Aarhus University, and were video-recorded and transcribed fully before coding.

### Analysis

**Figure 1.**
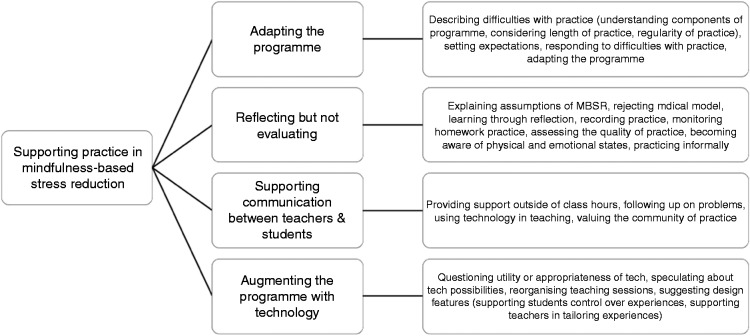
Supporting home practice: four themes and subthemes.

Thematic analysis^25^ was used to identify key themes and subthemes within the data. An exploratory inductive approach was considered appropriate because of the novelty of the area being investigated. Initial sets of codes were produced independently by the first and senior author, each going through the open coding process twice. Initial codes were refined through discussion between the coders and a consensus code book was established. The new set of codes was then reapplied to the data by both coders, again refining as appropriate. At this point, a meeting was held in which codes were collapsed into themes. One coder then double-checked the fit of the themes to the data through close reading. Figures of the themes and codes were merged to a single graphic model (Figure 1).

#### Reflexivity

In qualitative research, the subjectivity of the researcher is considered a valuable tool in understanding and interpreting data. There is no attempt made to achieve objectivity. Nonetheless, steps must be taken in order to ensure that findings of research do not simply reflect the existing assumptions or biases of researchers.^26^ Reflexivity is a process through which the researcher engages actively and continuously in critical reflection in relation to the knowledge produced by the work, and the methods through which it was produced. At the outset of the project, the researchers had significant concerns over the appropriateness of engaging with technology while practising mindfulness, but were also hopeful about the potential for using technology to remind people to practice. These positions were noted and were referred to throughout the process of data analysis, to avoid simply searching for data that supported our existing assumptions.

### Ethics

All participants provided written informed consent before taking part in this study. We followed the procedures of the local ethics committee and the project was registered with the Danish Data Protection agency (AU-2016-051-000001).

## Results

Results suggest four overarching themes that describe the experiences of teachers and students in relation to home practices in MBSR, as well as their thoughts and concerns regarding how technology can support those experiences. The four themes were adapting the MBSR programme when home practice is difficult, reflecting but not evaluating, supporting communication and understanding between teachers and students, and augmenting the course with mobile technology (see Figure 1). Participants from the teacher group were labelled T1–T5, participants from the student group were labelled S1–S5.

### Adapting the MBSR programme when home practice is difficult

The first theme describes both the difficulties that students experience in completing a demanding schedule of daily exercises during the MBSR course, and the various ways that teachers respond upon identifying student difficulties.

### Understanding difficulties with home practice

Both teachers and students spent large portions of the interviews discussing the difficulties with engaging regularly in home practice. Students in MBSR are expected to engage in a set of regular exercises in order to achieve benefit, and teachers (e.g. T1) advise students to set one regular time slot in which to practices every day. S1, S2 and S3 stated that they did not practice every day and that they should have. Two students (S2 and S3) regularly practiced at the same time slot each day (or at least each day that they practiced). S1 did not have a set time slot, but felt that, in hindsight, she would have liked to. S4 suggested that a set time would have been negative for her, and that she enjoyed the freedom to fit the exercises within her day. Given these reports, a question remains as to whether flexibly scheduling exercises would increase or decrease engagement in MBSR home activities.

One of the teachers (T1) suggested that a significant problem may lie in the length of home practice sessions, which last 45 min to 1 h per day. However, this did not emerge consistently from the students’ discussions. Indeed, S2 and S3 suggested that the length of each practice time was a fine positive aspect of the course. S2 suggested that it took at least 20 min at the beginning of a session for her to engage fully with the practice, while S3 felt he needed practice, and regularly extended the sessions beyond 1 h. S4 reported that it was difficult to engage in long sessions, but this was due to the emotional difficulties involved in the task, rather than scheduling time.

Participants suggested that many of the assigned activities are inherently difficult or uncomfortable to do, either physically or emotionally, especially at the beginning of the programme. Participants reported an increased awareness of ‘my suffering, my pain, my nausea’ (S2), when undertaking meditation; ‘sometimes I think I’m in a good state but when I lie down I can feel I’m not’ (S4). Indeed, participants rarely described the home exercises as enjoyable, with a number of students suggesting that they ‘hate’ (S2) doing them, at least some days, although they often felt better afterwards.

One of the main difficulties highlighted by participants is the experience of uncertainty over whether they were doing the exercise correctly. Indeed, the teachers discussed moving students away from an initial tendency of evaluating their own performance. One teacher noted that with MBSR, improvements in well-being may take a while to be apparent, regardless of how much daily practice is undertaken (T1). It also takes time to gain an understanding of what is, and is not, appropriate during meditation; ‘If people report back for instance, “Oh, it doesn’t work for me because my mind is wandering all the time,” that is not an obstacle – that is just … “Well, so you are becoming aware of the present moment”’ (T2). Compounding this difficulty is the fact that trying hard to do the exercise ‘correctly’ is often self-defeating in mindfulness practices, ‘I focused on to do it right, instead of doing it with mindfulness’ (S1).

Home practice in MBSR comprises a number of different activities: yoga, sitting meditation and the body scan. Participants reported very different experiences across the different types of exercises. For example, S1 and S3 found the yoga exercises much easier, as they were less likely to be interrupted by others in the house. Yoga exercises look like ‘working out’ and signal ‘do not interrupt’, in a way that the other exercises do not. For instance, one participant described the misunderstanding from others in her household when she completed the non-yoga exercises, ‘my husband said “Was it a good rest?” No, it was not a good rest’ (S3). Conversely, S2 reported that the yoga was most challenging. Thus, ‘home practice’ is not one homogenous activity, but a number of different activities that may be experienced as enjoyable or aversive by different students.

### Responding to difficulties with home practice

Teachers spent considerable interview time discussing how they respond when they learn that a student on their course is not engaging in the prescribed amount of home practice. Student problems with engagement are typically addressed within weekly classes, ‘you use what the participants are sharing, to sort of normalise also, to say out loud [that] the others in this group have the same experiences’ (T4). In smaller classes (<20 students), teachers often begin the session by going around the room asking each student about their practices during the week. In larger classes (up to 30 students), teachers offer some time at the beginning of class when students can volunteer to discuss their challenges. When students report problems, teachers engage in a dialogue with students, for instance to encourage reflection on their motivation for starting the course, ‘I put it in a larger perspective, about your life’ (T3). Teachers also discussed using a structured enquiry process to understand the barriers disrupting engagement, which involves ‘going into dialogue and actually asking questions like, “So you've only been practising 1 day of these 7 days; would you wish that it was different? Would you like to practice every day?” “Yeah, yeah I would, but I just can't find the time”. “Okay, so you can't find the time; what is in the way of you practising?”’ (T2).

In discussing their responses to problems, all teachers first emphasised the importance of setting expectations regarding home practice before starting the programme. Teachers make it clear that benefits are achieved if students engage in the daily activities: ‘telling people that “if you are not sure that you are motivated, you are not sure that this is the right time, better not start”’ (T1), and this generally works as a motivator for students (T2). When problems arise, students are reminded of their previous commitment to doing the work of home practice, ‘like heightening their awareness. Why are they not doing the home practice? Because they signed up for the programmes and knew this would be required’ (T2).

### Adapting the MBSR programme in response to challenges

One of the most interesting tensions to emerge from interviews was the question of whether, and when, it is appropriate to adapt the home practice for students who are experiencing challenges. Three of the teachers (T3, T4, T5) suggested that they often tune home practices recommendations to the abilities and situations of students, ‘I say, “just do a little”. And I have personal talk with them. I think that’s very helpful’ (T3); ‘we work with the programme for each class, each course and each person. We will make it suitable’ (T4); ‘also if people are very uncomfortable sitting still, you can do the practice walking. Or if you have chronic pain, you can do the practice laying down with your feet up’ (T3).

Students also said that they often adapted home practices to suit themselves, rather than strictly following the assigned exercises. This included doing shorter sessions when they were having difficulties (S2), lying down when they became tired of doing sitting meditations (S2, S4), and picking and choosing the exercises that they preferred (e.g. yoga over body scan).

In contrast with the students, a number of the teachers strongly emphasised the importance of engaging with the programme of exercises as specified. MBSR is an evidence-based, standardised course, and if elements are changed, participants may not benefit as expected. Moreover, MBSR requires students to meet challenges in their lives through doing the exercises. Adapting the programme in order to avoid difficulties or challenges may undermine its usefulness, ‘we don’t compromise the programme. We don’t compromise – it is 45 minutes’ (T2).

I have just been in the group where people talked a lot about why they didn’t do the home practices and it was so much talk and nobody came to do the home practices! There was a lot of talk about ‘I didn’t do it because of this and this’, it was like a confession round, and we were just two people doing the homeworks![*sic*] But it didn't help them. It was like more talking about nothing (T1).

‘You don’t have to like it, just do it’ (T5).

### Reflecting on practice, but not evaluating

The second theme describes the value placed by both students and teachers on careful reflection on the experience of practising. In the MBSR programme, significant time is spent practising and then reflecting on what occurred during the practices (e.g. ‘What am I learning? What am I noticing?’). This theme covers participants’ and teachers’ discussions on whether and how the activity of recording and monitoring home practice can support reflection. The practice and the reflection are both essential: ‘people actually learn about mindfulness and about their lives basically, not just from experience, but from reflecting upon experience’ (T2).

Both teachers and students regularly emphasised the importance of increasing the awareness of one’s moment-to-moment experience. Participants emphasised that activities are designed to increase awareness of whatever physical and emotional state exists, ‘I found out it doesn’t have anything to do with mindfulness that you have to be relaxed, you just have to be. And feel’ (S1). Indeed, two participants (S3, S4) and one teacher emphasised that mindfulness can actually increase suffering in the short term, ‘It is both good and bad to enhance your awareness. Then you become more aware of both the good and the bad things’ (T2). However, both teachers and students distinguished increasing awareness and reflection from a more conscious, and evaluative process: ‘We tell the participants not to evaluate. To continue. To forget about the goals’ (T3). Teachers cautioned against facilitating students to fixate over whether their health (or their meditation, or their yoga) was improving or not. This may turn students' attention away from the present-moment awareness and acceptance that mindfulness practice is intended to promote.

Teachers mentioned that there are a number of different methods that can be used to encourage student reflection, but these were not universally implemented. For example, T3 asks students who are struggling with practice to write about that experience in a diary, and students can choose whether or not to share this with the teacher. T5 mentioned that students are asked to keep a logbook of their practices after the eight-week programme finishes, but that this might also be useful during the programme, ‘this is very simple and would just be registering how many minutes and maybe what did you notice’ (T5).

Both teachers and students saw advantages in recording practices in a more structured manner. For instance, this could provide a new way for teachers to know who is doing the exercises (T2, T3). One teacher also suggested that it could be helpful in increasing students’ own awareness, ‘To record “today I practiced for thirty minutes”, just so that they get a sense of how much or how little they practice. I think that would be helpful feedback’ (T2). One student suggested it could be helpful to look back and reflect on previous sessions (S1). However, a number of students disliked the idea of recording their practices more formally, citing a mismatch between the type of behavioural monitoring that they are familiar with (e.g. for fitness training) and the goals of mindfulness. S3 suggested that it took so long to find benefit from mindfulness that it would never show up meaningfully in a diary or other record. S3 also worried about whether recording and sharing experiences would lead to a competitive atmosphere between classmates. Finally, T1 summed up the general point saying, ‘it is good to know that they do practice. And not to measure every second minute whether it is working’.

### Supporting communication and understanding between teachers and students

One potential advantage of technology is in improving communication between teachers and students. To do so, it is important to understand how this currently takes place in MBSR. Our data suggests that the vast majority of communication takes place face-to-face in the weekly classes. However, there are situations where teachers make themselves available outside of class times. For example, some teachers said they were available for conversations directly after class (T3, T5). All teachers mentioned being available either via email or phone for students who have urgent issues.

Teachers discussed how they currently follow up on problems that arise. The most common method was to discuss the issue during the following week’s class, but teachers also mentioned using other methods. T1 made phone calls to people who missed class sessions. T3 sent emails to students after class. She also asked them to write about the experiences that they found challenging, and then read and discussed their responses, ‘I do a lot of writing in the email, I actually use that interaction that way’ (T3). T2 mentioned that having access to some form of record of students’ practices (either a record of regular practice, or some reflective writing) would be helpful in following up on problems,

it might be helpful actually, not to use it in any way, but just to know that one participant is not practising at all would be helpful because I think I would go to that person. I wouldn’t say ‘so you haven’t been practising’. I would go and say how are things going for you? (T2).

Apart from emails and phone calls, teachers and students reported using a variety of different technologies to support their courses. All teachers made audio recordings of the formal meditation practices, and one hosted the sound files on a website (T3; SoundCloud). Students are expected to (and prefer to) listen to these guides rather than generic guides. Interestingly, T3 reported noticing when students played files via SoundCloud,

I can see participants using it. And I can see I feel a joy when they use it so maybe that … I can follow which of the participants use it, and the days they are using and their name on it (T3).

Students did use a number of apps for guided meditations and some were aware of apps that could track the length of meditation practice.

Another form of communication that participants valued was peer-to-peer social support. More specifically, students appreciated the ‘community of practices’^27^ that develops as a class moves through the programme, learning from the course content and from each other’s experiences. All students suggested that they enjoyed meditating in the weekly group sessions, ‘In a group, I just felt a very strong connection with the other people in the room’ (S1). Students reported sharing, laughing, being touched by other people’s stories and experiences. Indeed, they suggested preferring this to practising at home on their own, ‘we remind each other we are here just now altogether doing this. It is so much easier then when you are at home’ (T1); and ‘I need to do it with others. It gives me something else, something deeper, to meditate with other people’ (S3). Indeed, many students reported missing the group when the eight-week programme finished.

### Augmenting the programme with mobile technology

This theme describes the reflections of teachers and students on how technology could change their experience of delivering or taking part in a course. Contrary to our expectations, teachers were generally excited about how new technologies could support and improve the delivery of MBSR programmes. Teachers suggested that information on student activity levels and sleep schedules could be useful to know, as general contextual information (T2). Teachers suggested that technology could help better identify when people are struggling, but are unwilling to talk about it (T1). Students suggested that technology could be used to organise and coordinate group meditation or yoga sessions beyond the face-to-face classes, or after the eight-week course ends (S4). There were also many specific suggestions for technologies, generally based around features of mobile phone apps, such as reminders to practice, a way of recording their motivation for participating and reading material for the course.

Many of these suggestions focused on ways of supporting students’ sense of control over the system. They were resistant to the idea of an app that would force them to work through the MBSR programme in an inflexible manner, and emphasised the importance of making features such as reminders optional (T5). Another suggestion was to allow participants to decide when they should get reminders to practice if they had missed some sessions, ‘Could it be individual? Maybe I would like to have it after 3 days? Maybe she would like to have after a week? Maybe you could decide yourself?’ (S1).

Teachers and students also emphasised the importance of supporting students’ personalisation of the technology. Teachers suggested that students could be able to leave inspirational quotes for themselves, and to personalise practice reminders with these quotes (T4), as well as movies, poetry and pictures. Teachers also expressed a preference for being able to edit and tailor any technology that they use with their course to suit the needs of a specific group or a specific context. They also suggested that it would be important to include their own personal guided practices for participants, ‘so that my participants could listen to me and not to [another teacher’s name], but to me’ (T3). Students also noted that they wanted to hear their own teacher’s voice (S1, S2), rather than a generic teacher’s voice, as a way of connecting back to the class experience.

Many concerns were also raised over how the introduction of new technology might interfere with or undermine the programme. For example, teachers were concerned with students becoming competitive (T3), or diligently trying to report good practice times rather than engaging honestly with the exercises (T4, T5). One teacher emphasised the importance of students’ autonomy, ‘And also it is up to the person themselves to do the practice so … we don’t check it’ (T1). While there may be opportunities to support participants using technology, teachers did not want to encourage inappropriate evaluation, or a shift in responsibility for undertaking the home practices. Finally, there were also teacher concerns about the implications of being able to view what participants did outside of class time. One suggested that it could add to their workload, ‘it would require more work and things to think about’ (T1). They also identified more complex difficulties that this could raise: could the teacher respond fairly to those who they knew were not practising? ‘And then … how can we as teachers go into the class not having an agenda’ (T4), and ‘it might create a bias beforehand now I have to pay attention to these people’ (T2).

## Discussion

The aim of this work was to understand the requirements for technology that can support participants in undertaking the at-home practice recommended within standard mindfulness-based interventions. To this end, we interviewed experienced mindfulness teachers and past course participants about a) their experiences of home practice and b) their beliefs about technology usage in these courses. We outline how teachers and participants address challenges in completing the assigned home practice. We reflect on several considerations for technology design for mindfulness interventions (major points presented in Table 1), and highlight areas where we see potential benefits, as well as concerns.

**Table 1.** Suggestions for design of technology to support mindfulness course participants. Suggestions are derived from (a) discussions with students and teachers about design of a mobile tools and (b) more general discussions around home practices in mindfulness-based stress reduction. We include quotes from students and teachers to illustrate each issue.

Theme	Specific issue	Illustrative quote from student/teacher	Potential technology-based Solution
*Adapting the MBSR programme Subtheme: Responding to difficulties with home practice*	Participants have questions about how to engage with their practices (Am I doing it correctly?)	T2: ‘If people report back for instance, “oh It doesn’t work for me because my mind is wandering all the time,” that is not an obstacle’	An FAQs section, answering common participant questions or concerns
*Adapting the MBSR programme Subtheme: Responding to difficulties with home practice*	Continuing practice after the end of the 8-week course is challenging	T5: ‘I also see the possibility of having this access after the course; they can go through the programme again half a year later, so they still have the possibility to go through the pdf files, the teachings’	Continued access to course materials, introduction of new ‘advanced’ post-course meditation guides
*Adapting the MBSR programme*	Participants undertake some forms of practice more than others	T2: ‘So did you do your home practice today? No? Okay, what prevented you from doing your home practice?’	Provide feedback on the number of each types of practice participants complete to increase awareness of obstacles
*Adapting the MBSR programme*	Some participants struggle to complete practice as assigned	T3: ‘If people are very uncomfortable sitting still, you can do the practice walking. Or if you have chronic pain, you can do the practice laying down with your feet up. So do some positions that changes can be helpful to be in the uncomfortable feeling … so I think we can set it up in that way’	Provide teachers with the possibility of giving shorter practice assignments or other adjustments
*Adapting the MBSR programme*	Teachers do not always know who is struggling	T2: ‘We don’t really know how they are doing. Because it is a lot of people in our classes, 30 people sometimes’	Integrate participants’ midway evaluations into an app
*Reflecting, but not evaluating*	Promoting participants’ reflections on their learning	T2: ‘People actually learn about mindfulness and about their lives basically, not just from experience, but from reflecting upon experience’	Diary function, to record experiences for future review
*Reflecting, but not evaluating*	Participants do not currently record home practice in a structured fashion	T2 : ‘I think it would be a great idea for them actually. To record today I practiced for thirty minutes, just so that they get a sense of how much or how little they practice. I think that would be helpful feedback’	Automated recording of number of minutes of practices, time of day
*Reflecting, but not evaluating*	Ensure participants are not evaluating the practices experience	T1: ‘It is good to know that they do practice. And not to measure every second minute whether it is working’	Design should not promote an evaluation of practice (i.e. a star for ‘practices complete’), but should promote non-judgemental awareness of practice patterns
*Supporting communication and understanding between teachers and students*	Teachers use diverse methods to communicate with students outside of class time	T3 ‘But we say that we are available [by] phone call or something … sending an email, set up a phone call after’	Provide a convenient means for participants to communicate with the teacher from within an app, e.g. through email or a messaging feature
*Supporting communication and understanding between teachers and students*	Teachers use diverse methods to provide audio guides	T3: ‘I have a thing to add; also my soundtrack is on a place called SoundCloud’	Provide a consistent, convenient means for participants to access practice guides
*Supporting communication and understanding between teachers and students*	Students missed the group setting after the end of the course	S4: ‘I miss the group now, I miss a group’S4: ‘You could say, “Saturday morning I am doing meditation does anyone want to join?” I think that is nice’	Provide a means to keep in touch with other participants from the class after the course has ended
*Augmenting the programme with mobile technology*	Reminders to practice	S1: ‘Could it be individual? Maybe I would like to have it after 3 days? Maybe she would like to have after a week? Maybe you could decide yourself?’	Allow participants to personalise their own reminder settings
*Augmenting the programme with mobile technology*	Supporting participants’ motivation to practice	T4: ‘If people could choose if they wanted to put in … sort of a saying for themselves or reminders that comes up “Hey, you want to do this because? Do you remember?”’	Include space for participants to note quotes, images, poetry to inspire practices
*Augmenting the programme with mobile technology*	Supporting discussions with participants who are struggling with practice	T1 ‘I think it could be useful, but as a student and as a teacher because then you can talk about it … if you don’t know that someone is not practising, then you can’t talk about it’	If teachers can view participants’ practice time, they will be able to discuss issues with participants that may otherwise not come to light
*Augmenting the programme with mobile technology*	Personalisation is important to participants	T5: ‘That is usually what we also do in the programme itself … like the teacher. Your teacher who is guiding you through the files’	Delivering a teacher’s own meditation guides in an app
*Augmenting the programme with mobile technology*	Personalisation (choices about accessing students’ data) is important to teachers	T1: ‘It would require more work and things to think about’	Teachers may not be able to take on additional work such as reviewing participants’ practice time. This should be optional
*Augmenting the programme with mobile technology*	A Key component of the course, the midway evaluation is time-consuming	T2: ‘But that Midway assessment, I am just thinking that could be part of that application because that is actually talk quite time-consuming, it is taking away the time to interact with people, and in a way a bit disturbing’	The midway evaluation could be included in the app, so that participants could complete it outside of class time

MBSR: mindfulness-based stress reduction

### Adapting the intervention

While teachers receive training on how to deal with situations where students are struggling with the schedule of home practices, they reported using a range of different methods to meet these challenges. While teachers acknowledged the importance of practice as one component of the course, they did not collect information on students’ behaviour in a systematic way. We suggest that mobile tools could help teachers to monitor student practice in real time, with a level of accuracy not currently possible with paper-based methods. This would support teachers in larger classes, where it may be difficult for them to address each student individually. In addition, mobile monitoring tools could support teachers in intervening, where problems are observed (e.g. by prompting a dialogue between teacher and student as to the difficulties faced).

The students often reported adapting the schedule of at-home practice sessions to suit their needs. A number of the students reported preferring some types of practice over others (e.g. yoga to sitting meditations) and did more of their preferred practices. A number of qualitative studies have reported similar differences in participant preferences across the formal exercises. For instance, patients with breast cancer preferred and engaged in more yoga than other exercises,^28^ while patients with Obsessive–Compulsive Disorder more frequently rated the body scan as more helpful than yoga.^29^ While the students reported adapting the practice schedule to best fit their own needs and preferences, there was no clear consensus among teachers as to whether this should be supported. Some teachers valued considering the needs of the individual students, while others more strongly emphasised that the programme is a standardised, validated treatment.

One solution might be to provide teachers with the flexibility to make decisions on whether students’ practices schedules should be adaptable on an individual or class basis (e.g. in providing shorter practices to those students who need them). Indeed, several recent reviews have discussed how adaptations to mindfulness-based interventions can be helpfully formatted to fit the population and context in question.^30,31^ There is balance between maintaining the integrity of the intervention, and ensuring its acceptability and accessibility, especially in a non-research community sample. For a mobile tool, an appropriate adaptation or personalisation for students may lie in allowing them to choose the frequency and timing of reminders to practice. However, other work testing a stress-management app suggested that daily notifications, compared to occasional notifications, promoted greater exposure to the intervention content.^32^ This would require testing in relation to MBSR. Another technology-based solution might be in showing participants the number and frequency of practices types (e.g. yoga) that they completed, to increase their own awareness of their preferences, and also if they are ‘missing’ one of the assigned practice.

### Reflecting but not evaluating

To be consistent with the principles of MBSR, technology solutions should help students to become aware of their own behaviour patterns, but not in evaluating these as either ‘good’ or ‘bad’. For instance, typical monitoring approaches (e.g. for physical activity) present rewards (stars, badges) for goal achievement. This type of approach conflicts with the fundamentals of mindfulness-based interventions. Both teachers and students cautioned against the use of metrics-based systems that might promote evaluation of behaviour or competition. It is appropriate to increase awareness of behaviour patterns, but not to introduce any design elements that might push students towards critical evaluation. Teachers identified an opportunity to support participants in reflecting on their experiences with home practice, for instance through the use of diaries.

### Communication

We identified a clear opportunity to facilitate communication between teachers and students through technology. Teachers outlined differing methods used to communicate with their students, such as email and phone calls, consistent with implementation guidelines, (see Kuyken W, Crane and Williams^33^), and different methods of providing home practice guides (via email or other websites). We suggest that providing a streamlined, integrated solution to teachers and students may support communication beyond face-face sessions. It was also unclear whether teachers were aware of the students’ adaptations (e.g. choice of one mindfulness practice over another). Again, technology solutions could allow students to communicate the adaptations that they make more readily. Finally, another type of communication that technology might support is peer-to-peer communication between class participants. Peer-to-peer support was noted as an important component of the in-class experience, consistent with other qualitative studies.^34^ Since our intention is to improve engagement with home practice, there may be potential in facilitating a social connection during at-home sessions (e.g. by alerting participants to others currently practising), to replicate some of the rewarding features of group practice.

### Augmenting the programme with technology

Students and teachers provided multiple suggestions for augmenting the MBSR programme with technology.

Both emphasised the importance of personalised content, such as the provision of teacher-specific meditation guides, and a way to record and return to each student’s motivation for coming to MBSR. Teachers could see value in being able to see students’ practice completion, as a way to open discussions around any difficulties and to better understand their students in larger classes. Both teachers and students reported concerns about recording practices (prompting evaluation or between-student competition, trying to reduce a complex behaviour to a simplified number), which should be considered in any technological design.

Teachers reported that the midway evaluation in MBSR (a part of the curriculum) was a valuable opportunity to understand student progress and challenges with practice. This led to the suggestion that was broadly agreed upon, that midway evaluations could be integrated with a mobile application. Teachers suggested that this would be of benefit, as it could save class time.

Finally, there are questions as to how teachers will deal with new potential information on participants’ listening time behaviour, and how this might change student/teacher interactions and student expectations. Design of teacher tools should avoid adding significantly to their clinical workload. Ultimately, teachers agreed that it would be of use to ‘test out’ what it is like to have access to information on participants’ practice time, especially if they can have control over whether or not to use it.

### Limitations

We acknowledge that our sample size was small. However, the teachers that we interviewed were highly experienced mental health professionals, with substantial clinical workloads. The teachers were a difficult group of participants to access, and we believe that the length of the interviews allowed us to reach a saturation point in our data. We also interviewed participants who had completed MBSR courses for a variety of significant health concerns. Additional participant samples should be recruited to examine the requirements of specific subsamples, for instance those participating in MBSR for sleep problems or pain, and those participating for psychological distress. There are inherent issues with focus groups, such as the tendency for certain types of socially acceptable opinions to emerge,^35^ but we believe we heard contrasting opinions from participants here. Our focus was limited to understanding formal home practice, but participants also undertake informal practices during mindfulness-based interventions, like bringing awareness to everyday activities (e.g. chopping vegetables, eating a meal). Finally, most of the research on home practice in MBSR and MBCT has focused on formal practice, and this is arguably more amenable to technology-based measurement and support.

## Conclusion

At-home formal practice is a key component of mindfulness-based interventions and their completion is linked to intervention effectiveness.^9^ As with other psychological interventions such as CBT, these home assignments can be challenging for participants to complete.^36,37^ We suggest that there is potential for technology to support participants in engaging with their mindfulness practice. However, to fully realise this potential, there must be careful consideration of how technology can be used in a way that is complementary to the fundamental principles of mindfulness-based interventions. To this end, we explored how to design technology tools to support home practice in mindfulness-based interventions, through interviews with experienced teachers and students. These discussions highlighted important ideologies in mindfulness training for software and digital developers to consider.

There are clear opportunities to support communication between teachers and students, and to provide teachers and indeed researchers, with information as to how their students are engaging with home practice. Other helpful tools could support students in reflecting on their experiences and in providing reminders of their motivation for undertaking the course. There are questions as to whether and how the course might be adapted in the face of student difficulties, and whether technology should support participants’ adaptations (e.g. allowing selection of their preferred formal practices exercises). Finally, we should not encourage students to evaluate their practice as good or bad (e.g. through features typical in physical activity technology design like ‘step goal achieved’), as this would be counter to the course aims. Rather, we should aim to promote non-judgemental awareness of behaviour patterns, as development of awareness is a core focus of mindfulness training.
